# Pharmacogenomics and the Yin/Yang actions of ginseng: anti-tumor, angiomodulating and steroid-like activities of ginsenosides

**DOI:** 10.1186/1749-8546-2-6

**Published:** 2007-05-15

**Authors:** Patrick Ying Kit Yue, Nai Ki Mak, Yuen Kit Cheng, Kar Wah Leung, Tzi Bun Ng, David Tai Ping Fan, Hin Wing Yeung, Ricky Ngok Shun Wong

**Affiliations:** 1 Department of Biology, Faculty of Science, Hong Kong Baptist University, Kowloon Tong, Hong Kong SAR, China; 2 Department of Chemistry, Faculty of Science, Hong Kong Baptist University, Kowloon Tong, Hong Kong SAR, China; 3 Department of Biochemistry, Faculty of Medicine, Chinese University of Hong Kong, Hong Kong SAR, China; 4 Angiogenesis & TCM Laboratory, Department of Pharmacology, University of Cambridge, Tennis Court Road, CB2 1PD, UK; 5 International Society for Chinese Medicine, A & C, 1^st ^floor, Block 2, University of Macau, Av. Padre Tomas Pereira, Taipa, Macao SAR, China

## Abstract

In Chinese medicine, ginseng (Panax ginseng C.A. Meyer) has long been used as a general tonic or an adaptogen to promote longevity and enhance bodily functions. It has also been claimed to be effective in combating stress, fatigue, oxidants, cancer and diabetes mellitus. Most of the pharmacological actions of ginseng are attributed to one type of its constituents, namely the ginsenosides. In this review, we focus on the recent advances in the study of ginsenosides on angiogenesis which is related to many pathological conditions including tumor progression and cardiovascular dysfunctions.

Angiogenesis in the human body is regulated by two sets of counteracting factors, angiogenic stimulators and inhibitors. The 'Yin and Yang' action of ginseng on angiomodulation was paralleled by the experimental data showing angiogenesis was indeed related to the compositional ratio between ginsenosides Rg_1_ and Rb_1_. Rg_1_ was later found to stimulate angiogenesis through augmenting the production of nitric oxide (NO) and vascular endothelial growth factor (VEGF). Mechanistic studies revealed that such responses were mediated through the PI3K→Akt pathway. By means of DNA microarray, a group of genes related to cell adhesion, migration and cytoskeleton were found to be up-regulated in endothelial cells. These gene products may interact in a hierarchical cascade pattern to modulate cell architectural dynamics which is concomitant to the observed phenomena in angiogenesis. By contrast, the anti-tumor and anti-angiogenic effects of ginsenosides (e.g. Rg_3_ and Rh_2_) have been demonstrated in various models of tumor and endothelial cells, indicating that ginsenosides with opposing activities are present in ginseng. Ginsenosides and Panax ginseng extracts have been shown to exert protective effects on vascular dysfunctions, such as hypertension, atherosclerotic disorders and ischemic injury. Recent work has demonstrates the target molecules of ginsenosides to be a group of nuclear steroid hormone receptors. These lines of evidence support that the interaction between ginsenosides and various nuclear steroid hormone receptors may explain the diverse pharmacological activities of ginseng. These findings may also lead to development of more efficacious ginseng-derived therapeutics for angiogenesis-related diseases.

## Panax ginseng

### Background

Ginseng, *Panax ginseng *C.A. Meyer, a precious Chinese traditional medicinal herb, has been known clinically used in China for thousands of years. The genus name '*Panax' *was derived from Greek. 'Pan' means 'all' and 'axos' means 'cure'. Literally '*Panax*' can be translated as 'cure-all' or panacea. The herbal root is named ginseng because it is shaped like a man. Actually the term 'ginseng' represents two Chinese ideograms: 'gin' (pronounced ren) refers to 'man' and 'seng' (pronounced shen) refers to 'essence' It is believed to embody man's three mythical essences – body, mind and spirit. Thus it is also referred to as the lord or king of herbs [1]. Its medicinal efficacy was first documented in *Shengnong Bencao Jing *and it was later summarized by Li Shizhen in *Bencao Gangmu *and *Zhongyao Zhi *(Chinese *Materia Medica*) by People's Health Publishing House, Beijing, published in 1596 and 1959 respectively [1,2]. In the 18th century, the effectiveness of ginseng was recognized in the West, and subsequently, a large number of investigations were conducted on its botany, chemistry, pharmacology and therapeutic applications [3-7]. Ginseng has been used as a general tonic or adaptogen for promoting longevity especially in the Far East, especially China, Korea and Japan [8]. Ginseng is now one of the most popular herbal medicines used nutraceutically with an annual sale of over USD 200 million.

Ginseng is a deciduous perennial plant that belongs to the Araliaceae family. Currently, twelve species have been identified in the genus *Panax *(Table 1) [9]. Among them, *Panax ginseng *C. A. Meyer (Araliaceae), cultivated in China, Korea, Japan, Russia, and the US, *P. quinquefolium *L (American ginseng), grown in southern Canada and the US and *P. notoginseng*, cultivated in Yunnan and Guangxi provinces in China, represent the three most extensively investigated species. The pharmacological and therapeutic effects of ginseng have been demonstrated to affect the central nervous system (CNS), cardiovascular system, endocrine secretion, immune function, metabolism, biomodulating action, anti-stress, and anti-aging [5]. Recently, there have been controversies concerning the usefulness of ginseng in cancer therapy. Most studies claimed that the pharmacological effects of ginseng are attributed to its bioactive constituents such as ginsenosides, saponins, phytosterols, peptides, polysaccharides, fatty acids, polyacetylenes, vitamins and minerals [10]. In this review, we focus on the recent advances in the studies of ginsenosides on the modulation of angiogenesis (i.e. formation of blood vessels) which is a common denominator of many diseases, such as tumor and cardiovascular disorders (e.g. atherosclerosis).

**Table 1:** Species of Ginseng belonging to the genus *Panax*.

**Scientific name**	**Common name (English name)**	**Geographical distribution**
*Panax ginseng *C.A. Meyer	Asian ginseng Orient Ginseng Chinese Ginseng Korean Ginseng Red Ginseng White Ginseng Tartary Ginseng Tartary Root Kirin Ginseng	Korea Japan China Russia Germany
*Panax japonicus *C.A. Meyer	Japanese Ginseng ChiKu	East, Middle-south, South and South Yunnan Japan
*Panax bipinnatifidus *Seem	Feather-leaf bamboo ginseng Feather-leaved bamboo ginseng	Hubei, China Nepal Eastern Himalayas
*Panax notoginseng *(Burkill) F.H. Chen	Sanchi Ginseng Tienchi Ginseng Sanchu Ginseng Notoginseng San-qi ginseng Tien-qi ginseng Yunnan ginseng	Yunnan, Guangxi Guangdong, China
*Panax omeiensis *J. Wen	Omei ginseng	Sichuan, China Nepal Eastern Himalayas
*Panax pseudoginseng *Wallich	Himalayan Ginseng	South Tibet Nepal Eastern Himalayas
*Panax quinquefolius *L.	American Ginseng Occidental Ginseng Canadian Ginseng	Northeast, North, East China Southern Canada America (from Maine to Minnesota, south to Florida and west to Oklahoma)
*Panax stipuleanatus *H.T. Tsai and K.M. Feng	Pingpien ginseng *Baisanqi* *Tusangi* *Yesangi* *Zhujie qi*	Southern Yunnan, China
*Panax trifolius *L.	Dwarf ginseng Groundnut	Ohio Pennsylvania Nova Scotia to Wisconsin and further south
*Panax wangianus *S.C. Sun	Narrow-leaved pseudoginseng	Sichuan, China
*Panax vietnamensis *Ha et Grushv.	Bamboo ginseng Vietnamese Ginseng	
*Panax zingiberensis *C.Y. Wu and K.M. Feng	Ginger ginseng Ginger-like Pseudo-ginseng	Yunnan, China

[References: 5, 8, 9, MULTILINGUAL MULTISCRIPT PLANT NAME DATABASE- and Ginseng: A Concise Handbook. Edited by James A, Duke. Reference Publications, Inc. 1989. Michigan, USA]

### Ginsenosides of ginseng

**Figure 1:**
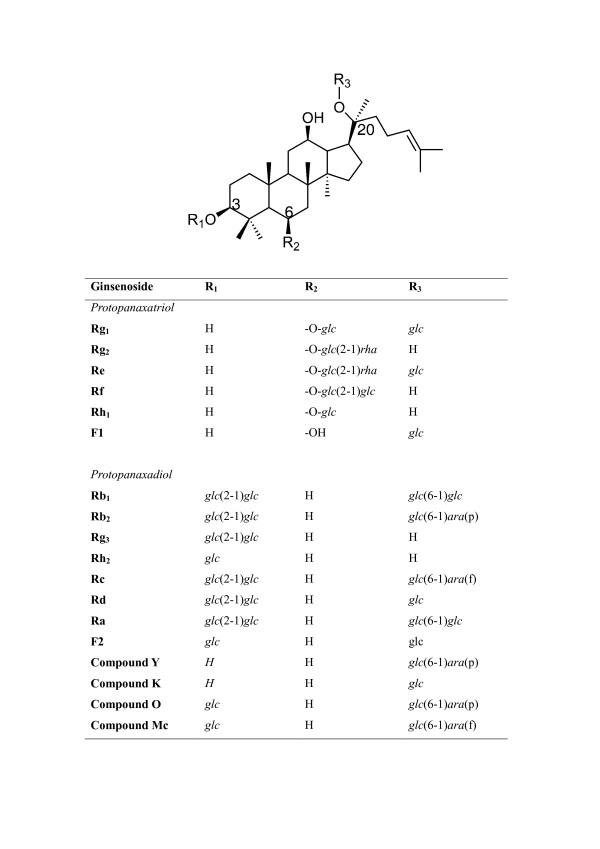
**The chemical structure of ginsenosides [6]**. *glc *= glucosyl (C_6_H_11_O_6_^-^); *rha *= rhamnosyl (C_6_H_11_O_5_^-^); *ara *= arabinosyl (C_5_H_9_O_5_^-^); p = pyran; f = furan.

The most prominent constituent of ginseng is a saponin glycoside known as ginsenosides (Rx) (Figure 1). Recent research indicates that most of the pharmacological effects of ginseng are attributed to ginsenosides [11]. In general, the contents of ginsenosides vary widely ranging from 2 to 20% depending on the species, age and part of ginseng, and even vary with the preservation or extraction method [11-13]. More than 30 ginsenosides have been isolated, and characterized from various *Panax *species [14,15]. In terms of their chemical structural characteristics, ginsenosides can be classified into three major categories, namely protopanaxadiols (PPD) (e.g. Rb_1_, Rb_2_, Rc, Rd, Rg_3_, Rh_2_), protopanaxatriols (PPT) (e.g. Re, Rf, Rg_1_, Rg_2_, Rh_1_) and the oleanolic acid derivatives. Ginsenosides have a steroid-like skeleton consisting of four *trans*-rings, with modifications from each other depending the type (e.g. glucose, maltose and fructose), number of sugar moieties and the sites of attachment of the hydroxyl group (e.g. C-3, C-6, or C-20) (Figure 1). Ginsenosides are amphipathic in nature. The hydroxyl (-OH) group of ginsenosides allows both interactions between the polar head of the membrane phospholipids and the β-OH group of cholesterol, while the hydrophobic steroid backbone can interact with the hydrophobic side chains of fatty acids and cholesterol. Indeed, these physiochemical interactions are greatly determined by the numbers and sites of polar hydroxyl groups on each ginsenoside. Moreover, ginsenosides have been shown to interact with numerous membrane proteins such as ion channels, transporters and receptors, which leads to a broad range of physiological activities [16].

## Angiogenesis

**Figure 2:**
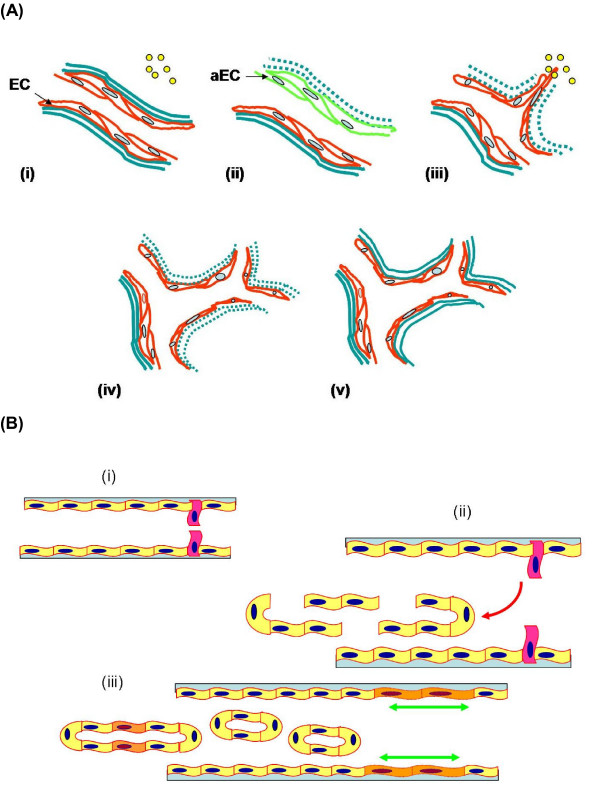
**The process of angiogenesis**. A) Sprouting angiogenesis: formation of blood vessels is a multi-step process, which includes (i) reception of angiogenic signals (yellow spot) from the surrounding by endothelial cells (EC); (ii) retraction of pericytes from the abluminal surface of capillary and secretion of protease from activated endothelial cells (aEC) and proteolytic degradation of extracellular membrane (green dash-line); (iii) chemotactic migration of EC under the induction of angiogenic stimulators; (iv) proliferation of EC and formation of lumen/canalisation by fusion of formed vessels with formation of tight junctions; (v) recruitment of pericytes and deposition of new basement membrane and initiation of blood flow. B) Non-sprouting angiogenesis – intussusceptive microvascular growth: it is initiated by (i) protrusion of opposing capillary walls towards the lumen; (ii) perforation of the EC bilayer and formation of many transcapillaries with interstitial core (red arrow); (iii) formation of the vascular tree from intussusceptive pillar formation and pillar fusion and elongation of capillaries (green arrows).

**Figure 3:**
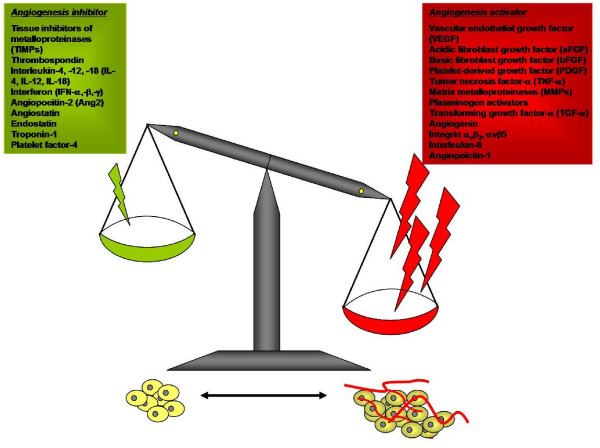
**The balance hypothesis of the 'angiogenic switch'**. Angiogenesis is tightly controlled by the balance of two sets of counteracting factors – angiogenic activators and inhibitors. The stability of 'angiogenic switch' determines the time of initiation of the subsequent angiogenic process. When there are more angiogenic stimulators than angiogenic inhibitors, as in the case of solid tumors, normal wound healing or female endometrial repair, the 'angiogenic switch' will be turned on and angiogenesis will proceed. Furthermore, during the process, each step is strictly mediated by the balance of different types of angiogenic stimulators or inhibitors. In some pathological cases, the 'angiogenic switch' remains in the 'ON' mode which leads to 'non-stop' formation of new blood vessels and ultimately many physiological disorders and diseases.

The term 'angiogenesis', first used by Hertig in 1935, refers to the formation of new blood vessels in the placenta [17]. Angiogenesis is a complex multi-step process which comprises activation, chemotactic invasion and migration, morphological alteration, proliferation, and capillary tube formation of endothelial cells (ECs) from pre-existing blood vessels (Figure 2). In this process, ECs have been shown to express all the information necessary to construct a vascular network. Under normal physiological conditions, most vasculature is quiescent, with only 0.01% of the ECs undergoing active cell division; thus angiogenesis is a relatively rare event that specifically occurs for a short and defined time period [18,19]. In addition, it is also tightly controlled by a relative balance of two groups of counteracting factors, namely angiogenic stimulators and inhibitors [20] (Figure 3).

### Sprouting and intussuceptive (non-sprouting) modes of angiogenesis

Sprouting angiogenesis consists of several consecutive steps with extensive interactions between soluble factors, extracellular matrix (ECM) and cells. As shown in Figure 2, during the early stage of angiogenesis, angiogenic factors such as vascular endothelial growth factor (VEGF) and fibroblast growth factor (FGF) emanate from conditioned cells (e.g. stromal cells, endothelial cells, blood) or surrounding tissues (e.g. ECM) to stimulate ECs [19]. These activated ECs secrete various types of enzymes including matrix metalloproteinases (MMPs), plasminogen activators (PA), gelatinase, collagenases and urokinases which bring about the degradation of basement membrane and ECM surrounding the parental blood vessels. Subsequently, the ECs migrate towards the angiogenic factors with the help of surface adhesion molecules or integrin receptors such as intercellular adhesion molecule-1 (ICAM-1), vascular cell adhesion molecule-1 (VCAM-1) and integrins α_v_β_3 _[21,22]. Upon the induction of various angiogenic factors, ECs proliferate and align in a bipolar mode to form capillary sprouts. Finally, these ECs form a lumen and a capillary loop through which the blood can flow.

Intussusceptive microvascular growth, a novel mechanism of blood vessel formation and remodeling, occurs by internal division of the pre-existing capillary plexus without sprouting. It is initiated by protrusion of opposing capillary walls into the lumen where a contact is created between the ECs. Then the endothelial bilayer is perforated and numerous transcapillary tissue pillars with an interstitial core are formed. An extensive vascular tree is formed from the subsequent intussusceptive pillar formation and pillar fusions [23-25].

### Molecular regulation of angiogenesis

Among the angiogenic regulators, VEGF and nitric oxide (NO) are the critical angiogenic stimulators. We have demonstrated that they are closely related to ginsenoside-mediated angiomodulation. Both VEGF and NO play a critical role in the regulation of physiological angiogenesis including embryogenesis, reproduction and wound healing. They have also been implicated in pathological angiogenesis associated with tumors or many cardiovascular disorders (such as atherosclerosis or ischemic injury). VEGF, which belongs to the VEGF family, is expressed in different tissues including the brain, liver, spleen and many cell types [26]. *In vitro*, VEGF not only acts as a survival factor and mitogen for ECs, but also induces the expression of many angiogenesis-related factors, including ICAM-1, VCAM-1, uPA, PAI-1, uPAR and MMPs. All these gene products are related to the increase of ECM degradation, migration and tube formation of ECs, whereas their effects on intussusception remain to be investigated [27-30]. The critical roles played by VEGF in embryonic vasculogenesis and angiogenesis have been demonstrated [31,32]. Inactivation of a single VEGF allele in mice resulted in embryonic lethality between days 11 and 12. The growth of *VEGF+/- *embryos was retarded and there were many developmental anomalies.

Endothelium-derived NO has been implicated in mediating a multiplicity of processes involved in angiogenesis. In fact, there are many angiogenic factors, such as VEGF, transforming growth factor β (TGF-β) and fibroblast growth factor (FGF), which can up-regulate the expression of endothelial NO synthase (eNOS), thereby inducing the release of NO [33-36]. VEGF- or bFGF-activated human umbilical vein endothelial cells (HUVEC) have been found to secrete NO on Matrigel substratum and subsequently form a tube-like structure, whereas such tube formating action is abolished by eNOS antagonist, N^ω^-nitro-L-arginine methylester (L-NAME) [37-39]. On the one hand, NO increases EC proliferation by acting as a survival factor and enhances migration by augmenting the expression of α_v_β_3 _and matrix-ECs interaction through the up-regulation of uPA [40-44]. Moreover, its vasodilating activity is the most prominent effect shown in the modulation of cardiovascular physiology [45]. On the other hand, disruption of the eNOS pathway can result in an impairment of normal angiogenesis, enhancement of tumor pathogenesis, and cardiovascular disorders [46-49].

VEGF and NO are working closely with each other in the modulation of angiogenesis, whereas NO is both an upstream and a downstream mediator or an effector of VEGF-dependent angiogenesis. Upon binding to its receptors, VEGF initiates complicated signaling cascades resulting in NO production and subsequent activation of ECs and smooth muscle cells in vessels, keratinocytes and macrophages in wounds, and tumor cells. Furthermore, through the activation of the PI3K/Akt pathway, NO can increase VEGF synthesis in a positive feedback manner [46,50-54].

### Angiogenesis in health and diseases

#### Normal physiology

In health, angiogenesis is under stringent control by an 'angiogenic switch' (Figure 3), and rarely occurs in adults except for embryogenesis, placentation, endometrial repair and wound healing [20,55]. In the latter case, formation of neovessels is necessary to sustain newly formed granulation tissues, in such a way that the ECs divide with a turnover rate of about 5 days, giving rise to a new microvascular network. During the menstrual cycle, however, angiogenesis occurs in corpora lutea and endometrium with rapid growth and regression [56-59]. Thus angiogenesis is normally in the quiescent state but is capable of both rapid activation and shutting down.

#### Pathophysiology

**Figure 4:**
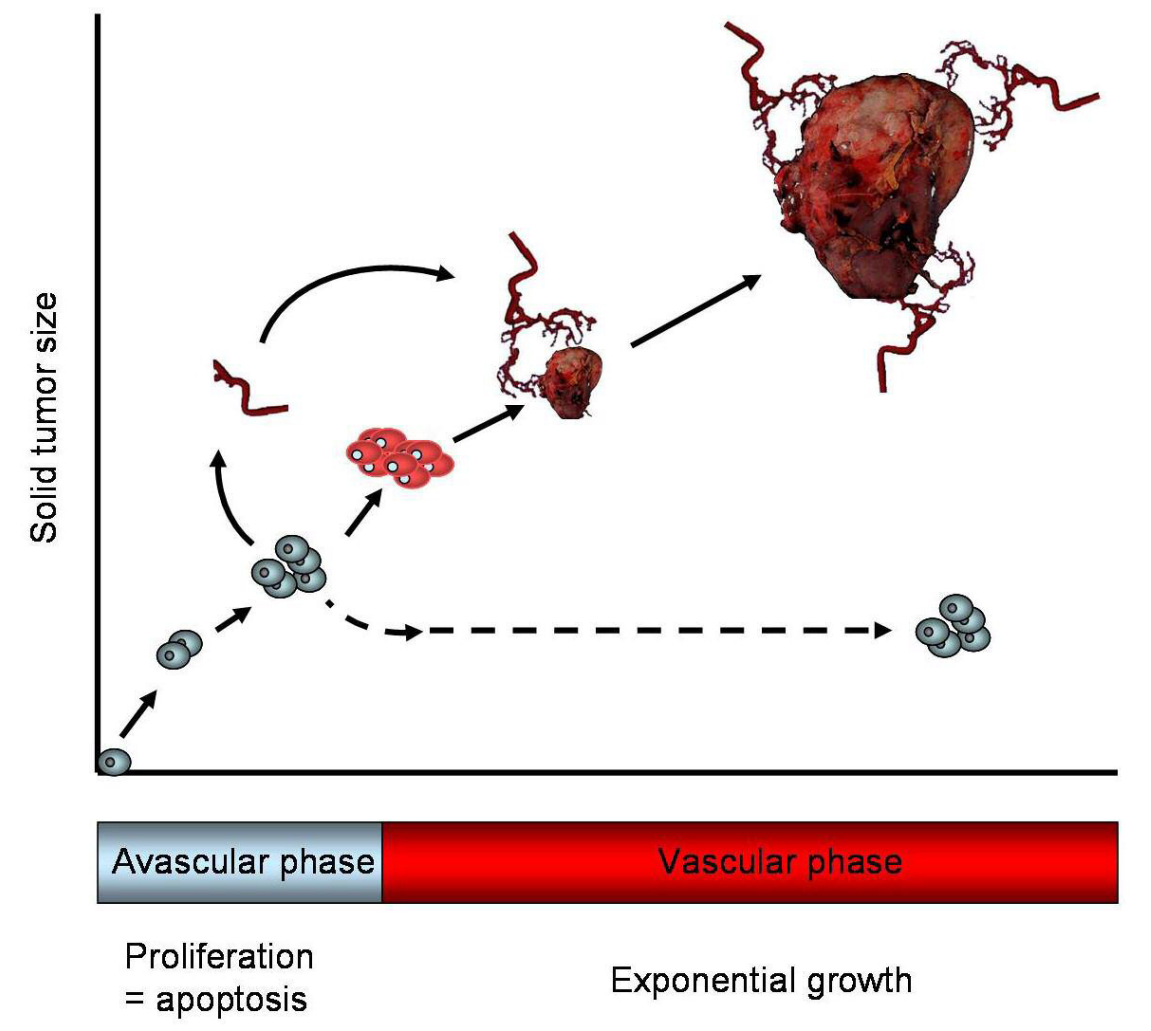
**A fundamental step in tumorigenesis – angiogenesis**. Angiogenesis is a critical step in the pathogenesis of solid tumors. Tumors remain in a dormant state (avascular phase) for a long time (up to several years), in which tumors keep their size within 1 – 2 mm^3^. When tumor progression starts, tumor cells secrete a large amount of angiogenic factors, mainly VEGF, to the surrounding tissues and blood capillaries. Once tumor angiogenesis is initiated, tumors enter a 'vascular phase' and become more aggressive. These newly formed blood vessels provide tumor cells with oxygen and nutrients for them to grow and for the initiation of metastasis.

Excessive angiogenesis has been defined as a prominent pathological feature of many diseases such as tumor, rheumatoid arthritis, atherosclerosis, psoriasis and diabetic retinopathy [60-63] (Table 2). During the early stage of tumorigenesis, tumors are usually not angiogenic (Figure 4). Since oxygen can only diffuse to around 150–200 microns from capillaries, solid tumors can only grow to 1–2 mm^3 ^autonomously at which stage they may exist for months or years without neovascularization (i.e. avascular phase). However, once tumor cells switch to the angiogenic phenotype (i.e. vascular phase), an extensive vascular network is constructed through sprouting or non-sprouting angiogenic mechanisms. The large amounts of angiogenic factors, mainly VEGF secreted by tumor cells or activated ECs, form a positive feedback on angiogenesis, leading to tumor growth and subsequent metastasis. [64,65]. A similar case has also been found in atherosclerosis, in which excessive angiogenesis enhances plaque growth in such a way to sustain perfusion, increase leukocyte exchange, deposition of proatherogenic plasma molecules and finally promote intraplague hemorrhage [66].

**Table 2:** Angiogenic diseases

**Diseases/disorders**	**Organ/organ system**
Atherosclerosis hypertension Vascular dementia Haemangioma Haemangioendothelioma Ischemic heart, limb	Cardiovascular system
Diabetic retinopathy Ischemic retinopathy Neovascular glaucoma Cancer	Ocular
Warts Kaposi's sarcoma Neoplasms Psoriasis Ulcer	Skin
Dysfunctional uterine bleeding Endometriosis Neoplasms Placental insufficiency Cancer	Reproductive system
Rheumatoid arthritis	Bones, joints
Ulcer Cancer Crohn's disease	Digestive system

An insufficient amount of angiogenic stimulators and/or an excess of angiogenic inhibitors can tip the balance resulting in inadequate or limited angiogenesis. Inadequate angiogenesis can also cause serious pathological outcomes such as stroke, Alzheimer disease, chronic wound, ulceration, ischemic coronary artery, critical limb ischemia, hypertension and hair loss [62,63]. In diseases such as ischemic coronary artery or limb ischemia, the functional blood flow is partially lost in certain organs or limbs. Poor blood circulation greatly prolongs the recovery period or even results in morbidity or death. A similar phenomenon has been found in the aberrant wound repair in diabetic and gastric ulcer [67,68].

## Angiotherapy: angiogenesis as a therapeutic target

Since the discovery in the early 1970s that tumor progression is angiogenesis-dependent, the concept of 'angiotherapy' has been hypothesized as a therapeutic strategy [69]. Ideally, this strategy could be used in the case of insufficient angiogenesis (e.g. heart ischemia) through the stimulation of neovessel formation by introducing angiogenic stimulators. An over-burst of uncontrolled angiogenesis (e.g. tumors) could be treated using angiogenic inhibitors to shut down the formation of neovessels [70-72] (Table 2). After thirty years, this idea became a reality when the first-generation anti-angiogenic drugs called Avastin (bevacizumab) and Macugen (pegaptanib sodium) were approved by the Food and Drug Administration in the US in 2004 and 2005 respectively for clinical application in cancer patients [73].

In fact, a large number of anti-angiogenic agents (e.g. thalidomide, TNP-470, endostatin, and angiostatin) are being tested or undergoing preclinical or clinical trials either alone or in combination with conventional therapies [74-76]. Most of these drugs target the ECs rather than tumor cells, thereby affecting different stages of angiogenesis. Therefore, they are less likely to cause bone marrow suppression, gastrointestinal symptoms or hair loss than conventional tumor therapies.

## Pharmacological actions of ginsenosides

### Anti-tumor effects of ginsenosides

In Asia, ginseng has been used as a medicinal herb for a long time to treat various ailments such as malignant diseases. Experimental studies from the past two decades have shown that both the anti-tumor activities and therapeutic effects of ginseng are mainly attributed to ginsenosides which induce cell death and inhibit metastasis.

#### Induction of cell death

Several patterns of eukaryotic cell death, namely apoptosis, autophagy, paraptosis, mitotic catastrophe and necrosis, have been recognized [77-79]. The process of cell death can be either caspase-dependent or caspase-independent. It is also known that mitochondria play an important role in the initiation of apoptosis. Different ginsenosides induce apoptosis through various signaling cascades. Rh_2 _and compound K (the metabolites of Rb_1_) have been shown to induce apoptosis in prostate cancer cells, ovarian cancer cells, neuroblastoma cells and A549 lung adenocarcinoma cells by activating caspase-3 and -8 [80-83]. Kim *et al*. further demonstrated that such apoptotic activation can be Bcl-2, Bcl-xL or Bax independent [84-87]. In human neuroblastoma (SK-N-BE) and rat glioma models, Rh_2_-induced apoptotic cell death has been implicated via protein kinase C (PKC) and reactive oxygen-dependent mechanisms [88-90]. The apoptotsis – inducing activities of other ginsenosides such as Rb_2_, Rc, Rg_3_, RS_4 _and IH901 (20-O-(beta-D-glucopyranosyl)-20(S)-protopanaxadiol, an intestinal bacterial metabolite of ginseng formed from Rb_1_, Rb_2 _and Rc, have also been observed in different human tumor cell lines [87-89]. These findings suggest that induction of tumor cell apoptosis by ginsenosides may be one of the mechanisms in the elimination of tumor cells.

#### Anti-proliferation

Inhibition of cell cycle progression has also been implicated as a chemopreventive mechanism of ginsenosides. Rh_2_, Rg_3_, IH901 and PD have been shown to arrest the growth of human tumor cell lines such as A549 lung tumor cells, LNCaP and PC3 prostate carcinoma cells, U937 leukemia cells, SK-HEP-1 hepatoma cells and HeLa cells [80-83,91]. Mechanistically, Rh_2 _has been shown to arrest the cell cycle at the G1/G0 phase, and to prolong the S-phase in intestinal (Int-407 and Caco-2) cells and SK-HEP-1 hepatoma cells [92-94]. It also induces cell arrest by down-regulation of cyclin/Cdk complex kinase activity, inhibition of E2F release in MCF-7 breast carcinoma cells, or by modulation of three modules of MAP kinases in prostate carcinoma cells [81,95].

Active growth of tumor cells can also be attenuated by induction of terminal differentiation and this approach has been developed as one of the strategies in cancer treatment [96]. Previously, Zeng and Tu showed that Rh_2_-treated hepatocarcinoma (SMMC-7721) cells exhibited the morphological characteristics of mature cells. Results showed that Rh_2 _can reduce telomerase activity which affects transcriptional activity in cells and facilitates both cell differentiation and cell arrest [97,98]. Moreover, Rh_2_-induced cell differentiation has also been found in B16 melanoma cells and F9 teratocarcinoma cells [99,100]. After Rh_2 _treatment, B16 cells resembled epithelioid cells morphologically and the cells were arrested at the G1 phase. *In vitro *induction of differentiation of F9 cells was shown to involve the glucocorticoid receptor (GR). In the presence of a glucocorticoid antagonist (RU486), Rh_1_- or Rh_2_-treated F9 cells did not differentiate into endoderm-like cells. These hypotheses were further corroborated in the gel mobility shift assay, in which the glucocorticoid responsive element (GRE) was specifically detected, mainly in the nuclear extract of ginsenoside-treated F9 cells. Concomitantly the results were also recorded in the luciferase-reported gene assay.

#### Anti-invasion and metastasis

Cancer metastasis is a complex process involving angiogenesis and cell-cell interactions. Enzymes such as matrix metalloproteinases are known to play an important role in tumor invasion, metastasis as well as initiation of angiogenesis. Fujimoto *et al*. recently demonstrated that the invasiveness of the endometrial cancer cell lines HHUA and HEC-1-A was inhibited by treating the cells with ginsenoside Rb_2 _[101]. The inhibitory effect is due to suppression of MMP-2 expression. The association between down-regulation of MMP expression and reduction of invasiveness was also demonstrated in a highly metastatic HT1080 human fibroblast cell line. Purified ginseng components, PD and PT, down-regulated the expression of MMP-9 in HT1080 cells [102]. By contrast, the expression of MMP-2 was not affected by PD and PT. Whether the observed differential inhibition of MMP-2 and MMP-9 expression in these two groups of cells is cell type- or ginsenoside-dependent remains to be elucidated.

In addition, the anti-invasive effects of Rb_2_, 20(S)- and 20(R)-Rg_3 _have been further demonstrated by Shinkai *et al*. [103] and Mochizuki *et al*. [104]. Rg_3 _was found to significantly inhibit *in vitro *invasion of rat ascites hepatoma cells (MM1), B16FE7 melanoma cells, human lung carcinoma (OC10) and pancreatic adenocarcinoma (PSN-1) cells. Intravenous (10 μg/mouse) or oral (100–1000 μg/animal) administration of both Rg_3 _and Rb_1 _inhibited lung metastasis of B16-BL6 melanoma and colon 26-M3.1 carcinoma in mice [103-105]. Furthermore, Rg_3 _greatly reduced the volume or weight of tumors in xenotransplanted (e.g. Lewis lung carcinoma, human breast infiltrating duct carcinoma, human gastric tumor and B16-BL6 melanoma) or chemical-induced (e.g. hepatocellular carcinoma) tumor models [106-109].

#### Effects of ginsenosides on multi-drug resistance

The development of multi-drug resistance (MDR) is a major problem in cancer chemotherapy. Agents that can enhance the accumulation of chemotherapeutic drugs in tumor cells by targeting the MDR proteins is a novel approach to overcome this problem [110-112]. In an acute myelogenous leukemia cell model, PPT ginsenosides were found to exert a chemosensitizing effect on P-glycoprotein (Pgp)-mediated MDR leukemia cells by increasing intracellular accumulation of daunorubicin and doxorubicin [113]. The effect on the reversal of drug resistance was due to competitive binding of ginsenosides such as Rg_3 _with Pgp, thereby blocking drug efflux [114]. In other MDR leukemia cells such as daunomycin or vinblastine-resistant leukemia cell models, various ginsenosides including 20(S)-PPT, Rh_2 _and compound K were found to greatly enhance the cytotoxicity of anti-cancer drugs in a range of 2- to 46-fold through the blockage of drug efflux [115]. Moreover, other ginsenosides including Rc and Rd reduced the efflux pump activity in lymphoma cells [116]. Recently, we also demonstrated that ginsenosides could exert the chemosensitizing effect through direct interaction with breast cancer resistance protein (BCRP), but not Pgp in MCF-7 cells. Among the tested ginsenosides, Rh_2_, PPD and PPT have been found to increase the cytotoxicity of mitoxantrone (MX), a potent anti-tumor drug, to human breast carcinoma MCF/MX cells which overexpress BCRP. The potency order of ginsenosides to inhibit BCRP is PPD > Rh_2 _> PPT. Neither Rg_1 _nor Rh_1 _was observed to inhibit BCRP or Pgp, whereas Rg_3 _was shown to be mild inhibitors. Previously, the C-6 substitution was hypothesized to confer anti-tumor activity. These findings suggest that the C-6 substitution is also important to the BCRP-inhibitory activity of ginsenosides. In addition, the two glucose substitutions at C-3 position such as Rg_3 _nearly abrogate its effect to inhibit BCRP. Our findings indicated that the inhibition of MX efflux as mediated by BCRP and the enhanced uptake of MX is correlated to the attachment of the hydroxyl group and sugar substitution at different positions of the steroidal skeleton of ginsenosides [117].

#### A new class of anti-tumor drugs: ginsenosides Rg_3 _and Rh_2_

Both Rg_3 _and its metabolite form Rh_2 _have emerged in Mainland China and Taiwan as anti-cancer drugs in the form of capsules (e.g. 'Rg3 *Shenyi Jiaonang*' and 'GOOD LIFE ginsenoside Rh2 capsule'). Rg3 *Shenyi Jiaonang *suppresses tumor angiogenesis and prevents adhesion, invasion and metastasis of tumor cells [118]. Rh_2 _as an adjuvant agent was also tested in the nude mouse model with human ovarian cancer cells transplanted. In the presence of Rh_2_, cisplatin could significantly inhibit tumor growth *in vivo *and prolong survival time. Neither Rh_2 _nor cisplatin alone could inhibit tumor growth [119]. It was shown that chemotherapy supplemented with Rh_2 _is 60% more effective than chemotherapy alone. It could also mitigate the adverse effects of hair loss, anemia, nausea, vomit and poor appetite following chemotherapy or radiotherapy [120].

Both Rg_3 _and Rh_2 _are extracted from red ginseng which is processed by steaming and drying. During the process, the malonyl group at the C-6 is released and the glycosyl moiety at C-20 is partially detached to generate Rh_1_, Rh_2 _and Rg_3 _through deglycosylation similar to the deglycosylated product, compound K generated from the metabolic transformation of ginsenoside Rb_1 _by intestinal bacteria [121,122]. However, whether all of the ginsenosides generated by such post-treatment of white ginseng have similar anti-tumor effects are still not known.

### Anti-angiogenic effects of ginsenosides

Various ginsenosides including Rg_3_, Rb_2 _and compound K demonstrated anti-angiogenic activity in different tumor models. Among them, Rg_3 _is the most extensively investigated. It exerts an anti-angiogenic action in different animal models when administered alone or in combination with other conventional chemicals. In those animal models which have been orthotopically implanted with different tumor cells such as human breast infiltrating duct carcinoma, ovarian carcinoma SKOV3 cells, B16-BL6 melanoma cells and colon 26-M3.1 carcinoma, Rg_3 _or Rb_2 _was found to inhibit angiogenesis *in vivo*. A significant reduction of intra-tumoral microvessel density (MVD), VEGF mRNA and VEGF protein levels in tumor tissues and sera of the tumor-bearing animals was observed [104-106,122]. Similar results were also reported when Rg_3 _was used in combination with a low dose of cyclophosphamide in bearing mice Lewis lung carcinoma [109]. These data indicate that one of the mechanisms of anti-metastatic effect of ginsenosides is probably related to suppression of tumor-induced angiogenesis [104].

**Figure 5:**
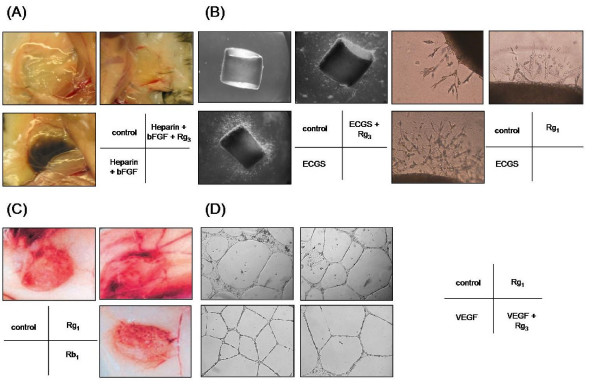
**Angiogenesis assays**. Angiogenesis is a multi-step process, Different types of *in vitro*, *in vivo *or *ex vivo *bioassays have been designed to mimic the various steps of angiogenesis. (A) *In vivo *Matrigel Plug assay: liquid form Matrigel (500 μl) containing growth factor (e.g. bFGF) and/or ginsenoside is injected into the abdominal region of C57/BL mice subcutaneously. The Matrigel will solidify at 37°C and form a solid 'plug'. After 5 days of incubation, the mice are sacrificed and *in vivo *angiogenesis including endothelial cell invasion, migration and formation of neovessels can be examined [124]. (B) *Ex vivo *rat aortic ring sprouting assay: rat aortic fragments one millimeter in length are embedded in Matrigel and cultured in the presence of growth factors (e.g. endothelial cell growth supplements – ECGS) and/or ginsenosides. The extent of endothelial sprouting from the aortic fragment can clearly indicate the angiogenic properties of ginsenosides [124]. (C) *In vivo *sponge implantation assay: a sterile polyether polyurethane sponge (170 mm^3^) containing ginsenosides is inserted into the abdominal region of Balb/c mice. After incubation for 15 days, the animals are euthanized and neovascularization is examined as indicated by the growth of vessels in the granuloma tissue [129]. (D) *In vitro *tube formation assay: endothelial cells are seeded on the Matrigel and subsequently incubated in medium containing growth factors (e.g. VEGF) and/or ginsenosides. Endothelial cells will rearrange and alight into a tubular structure. Angiogenic properties of ginsenosides can be reflected from the number of tubes, branching points or tube area.

In a recent study, we demonstrated that 20(R)-Rg_3 _(1–10^3 ^nM) was able to inhibit HUVEC proliferation, VEGF-induced chemoinvasion and tubulogenesis *in vitro *in a dose-dependent manner. In a Matrigel plug assay, 20(R)-Rg_3 _(600 nM) was able to reduce the hemoglobin content by 5-fold when compared with the positive control containing bFGF and heparin. The extent of microvascular sprouting was inhibited dose-dependently by 20(R)-Rg_3 _in the *ex vivo *organtypic cultures of rat aortic ring fragments (Figure 5). Basement membrane degrading enzymes, especially MMP-2 and -9, are secreted by activated ECs (or even tumor cells) throughout the angiogenic process. They have been demonstrated to play a critical role in cell invasion and migration, as well as angiogenesis. Our data further demonstrate that the anti-angiogenic effect of 20(R)-Rg_3 _was probably related to reduction of expression and activities of MMP-2 and -9 [124]. By using DNA microarray, 20(R)-Rg_3 _was found to inhibit the protein expression of angiogenic factors via several target genes (e.g. VEGF, bFGF and MMP-2) in both human lung adenocarcinoma cell line A549 and HUVEC304 cells [123]. Current research findings, indicating that some PD type ginsenosides (e.g. Rg_3 _and Rb_2_) exhibit potent anti-tumor and anti-angiogenic activities i*n vitro *and *in vivo*, have far-reaching implications for future development of ginsenosides as an anti-tumor medicine.

### Angiogenic effects of ginsenosides

In contrast to the anti-angiogenic effects of ginsenosides such as Rb_1 _and Rg_3_, another group of ginsenosides, represented by the panaxatriols Rg_1 _and Re, have been found to be angiogenic. Rg_1 _increased HUVEC proliferation, migration and tube formation in a dose-dependent manner [125-128]. We further demonstrated its angiogenic effects using the *in vivo *Matrigel plug and *ex vivo *aortic ring sprouting models (Figure 5). Histological evaluation of the Matrigel implants indicated that functional neovessels were formed as induced by Rg_1_. The *in vivo *data collected seven days after the implantation of genipin-fixed porous acellular bovine pericardium (same as extracellular matrices), which was dip-coated with Re or Rg_1_, indicate that the density of neovessels and tissue hemoglobin content inside the matrices were significantly increased by both ginsenosides. Furthermore, vascularized neo-connective tissue fibrils were found to fill the pores in the matrices loaded with Re [125]. Similar angiogenic activities were also observed in the above assays when Rg_1 _was used [126]. Interestingly, ginsenoside-induced angiogenesis was found to be comparable to or even better than bFGF-induced angiogenesis *in vivo*. These data suggest that both ginsenosides are very potent angiogenic agents and may potentially be useful in therapeutic angiogenesis in tissue-engineering strategies

Ginseng contains two groups of ginsenosides with activity in the modulation of angiogenesis. These observed counteracting effects can be interpreted by the Yin/Yang theory in traditional Chinese medicine (TCM). In fact, we have, in our previous publications, demonstrated strong scientific evidence supporting the ancient Yin/Yang theory [129]. Administration of Rg_1 _or Rb_1 _alone was shown to exert counteracting effects in angiogenesis. Rg_1 _alone promoted functional neovascularization in the polymer scaffold in Matrigel implant model and the chemoinvasion of HUVEC. By contract, Rb_1 _exerted inhibition in both. Moreover, we found that ginseng extract reconstituted with a defined ratio of Rg_1 _and Rb_1 _could alter the angiogenic outcome. In an *in vivo *scaffold implant neovascularization model, administration of an extract with a higher concentration of Rg_1 _than Rb_1 _(Rg_1_:Rb_1 _= 5:2) resulted in the induction of significant angiogenesis in the implant compared with control implants. In contrast, overabundance of Rb_1 _(Rg_1_:Rb_1 _= 2:5) inhibited Rg_1_-induced neovascularization [130]. These counteracting actions manifested the logic of Yin/Yang theory of TCM, which advocates that everything has opposing Yin and Yang aspects, and these aspects are reciprocally regulated and inhibited by each other in such a way that a continuous state of dynamic balance is maintained. The contents of ginsenosides in *P. ginseng, P. quinquefolius *and *P. notoginseng *were then measured by an HPLC method. The data show that *P. ginseng *has a higher ratio of Rg_1 _to Rb_1 _(0.51 – 2.08), while *P. quinquefolius *has a lower ratio (0.07 – 1.4) [131,132]. These findings are also consistent with the TCM's attributes of 'hot' (i.e. stimulating) properties of *P. ginseng *and 'cool' (i.e. calming) properties of *P. quinquefolius*.

### Functional genomics approach on the mechanistic study of ginsenoside

Functional genomics refers to the functions and interactions of genes towards a holistic view of the biological system in terms of gene expression and proteins. It focuses on the dynamics of gene transcription, translation and protein-protein interactions, instead of the static views of genomic information (e.g. gene sequences). It can be achieved by means of transcriptomics (differential expression of genes), proteomics (the study of total protein complement of a genome), phosphoproteomics and metabolomics studies (the study of the entire metabolic content of a cell or an organism). In general, the functional genomics approach is accompanied by high-throughput technology platforms such as the DNA microarray (i.e. DNA chip) technology and two-dimensional gel electrophoresis (2-DE) coupled with mass spectrometry for characterization of the abundant gene and protein products. The chip-based microarray allows a quantitative parallel assessment of gene expression and facilitates the study of complicated drug actions at the molecular level. This rapid increase in functional genomic-based drug studies enables an early and more accurate prediction and diagnosis of disease and disease progression. Understanding of the individual responses to drugs will have implications for their use and development by the pharmaceutical industry.

This approach was applied in the mechanistic study of ginsenoside Rg_1 _on HUVEC [127,133]. Our microarray data indicated that ginsenoside Rg_1 _could up-regulate a set of genes related to cell adhesion, migration and cytoskeleton such as RhoA, RhoB, IQ-motif-containing GTPase-activating protein 1 (IQGAP1), calmodulin (CALM2), Vav2 and laminin-α4 (LAMA4) in HUVEC. These proteins interact with one another in a hierarchical cascade pattern in modulating cell architectural dynamics. As we observed in the tube formation assay, HUVEC undergo rapid migration and morphological differentiation to form tubular structures by involving a dynamic assembly and disassembly of cytoskeletons. RhoA and RhoB, which belong to Rho family GTPases, have been found to regulate cell-cell adhesion and hence the motility through interfering with membrane ruffling, stress fiber formation and E-cadherin-mediated cell-cell adhesions [134-142]. The activities of GTPases are regulated by means of a molecular switch; cycling of the GDP-bound form (inactive state) and GTP-bound form (active state). As demonstrated by Bustelo *et al*., such transformation can be activated by guanine nucleotide exchange factors (Rho-GEFs) [143,144]. Vav2, one of Rho-GEFs, can increase the exchange of bound-GDP for a GTP molecule and translocate the Rho GTPases to the plasma membrane for interaction with its Rho effectors such as IQGAP1 [145]. Moreover, Vav2 can also affect cadherin-mediated cell-cell adhesion by binding with p120 catenin leading to formation of lamellipodia and membrane ruffling [146-148]. IQGAP1 acts as a negative regulator in E-cadherin-mediated cell-cell adhesion by interacting directly with β-catenin and the cytoplasmic domain of cadherin [149,150]. Over-expression of IQGAP1 is believed to compete with α-catenin for the same binding site on β-catenin and displace α-catenin from the β-catenin-α-catenin complex which disrupts the association of the complex with F-actin. Such cytomechanical modifications can subsequently cause the reduction of E-cadherin-mediated cell-cell adhesion that accounts for the changes in cell morphology and migration [151]. Moreover, laminins, which are the trimeric basement membrane glycoproteins, have been found to participate in the formation and differentiation of tubular structures of HUVEC *in vitro and *to provide a structural link between the ECM and the actin-based cytoskeletal system of cells [152-156]. Undoubtedly, microarray technology provides a high throughput platform for the elucidation of drug mechanism in study models.

On a different front, a proteomic study was carried out by Ma *et al*. to elucidate the cardiovascular protective role of Rg_1 _on ECs [133]. The data show that protein expression of MEKK-3, reticulocalbin, phosphoglycerate, 6-phosphogluconolactonase, zinc finger protein, NSAP1 protein, recombination-activating protein was increased, while that of eNOS, and mineralocorticoid receptor (MR) was decreased upon tumor necrosis factor-α (TNF-α) stimulation of HUVEC. However, Rg_1 _restored the expression of these proteins to the normal level. Western blotting and RT-PCR further validated that NO production in such TNF-α induced condition was correlated to the increase of eNOS mRNA and protein expression. They suggest that the increase of NO is related to the protective role of Rg_1 _on TNF-α stimulated HUVEC.

## Ginsenosides and steroids

### Steroid hormones and vascular homeostasis

A critical function of the vasculature is to provide a nutritional supply to tissues and organs. In general, proper vascular homeostasis is controlled by capillary permeability modulation, vasodilation and angiogenesis [157-159]. All these activities can govern the rate of material exchange, transportation and the site of distribution so as to meet the physiological needs of the human body. Steroid hormones, such as glucocorticoids, estrogens, progestagens, mineralocorticoids and androgens, play an important role in such vascular modulation at the molecular, cellular and even systematic levels [160-164].

Among the various steroid hormones, estrogen is the most promising example used to elaborate the maintaining and protective roles of steroid hormones on vascular homeostasis and cardiovascular disorders. The growing evidence implicated that estrogen reduces the risk of atherosclerosis by (1) decreasing the serum levels of both total and low-density-lipoprotein (LDL) cholesterol, while raising those of high-density-lipoprotein (HDL) cholesterol and triglycerides; (2) interfering with the inflammatory processes (e.g. monocyte adherence and trans-endothelial migration on the endothelium) in the vasculature; and (3) acting as an antioxidant [165-168]. Furthermore, estrogen is also a potent vasodilator, and hypotensive agents that can induce vascular relaxation by producing an increase of endothelium-derived vasodilators (e.g. NO). Mechanistic studies clearly indicate that while estrogen binds with its corresponding steroid hormone receptor – estrogen receptor (ERα, and ERβ), it may undergo either or both genomic and non-genomic actions inside the cells. Through the conventional pathway, activated-ER induces the gene transcription of eNOS, thereby increasing NO generation. Alternatively, through triggering the intracellular signaling (non-genomic) pathway, phosphorylated estrogen-bound ER (ligand-activated ER) has been found to induce rapid endothelial NO release via the PI3K-Akt-dependent pathway in HUVEC [169,170]. Various ginsenosides have been shown to exhibit an estrogen-like activity by acting as a functional ligand of ER [171-174].

### Ginsenosides-mediated steroid-like activities

**Figure 6:**
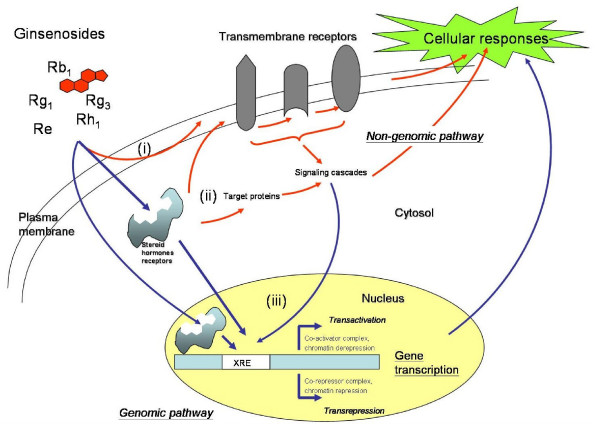
**Schematic overview of ginsenosides-mediated genomic and non-genomic pathways**. Ginsenosides possess a steroid-like skeleton composed of four *trans*-rings with different degrees of glyco-substitution. They are amphipathic in nature and can exhibit their actions at different cellular locations; such as the plasma membrane, cytosol and nucleus. Through the non-genomic pathway (indicated by red arrows), (i) they can initiate their actions by binding with the transmembrane receptors (e.g. ATPase pump, ion transporters and channels, voltage-gated channels and G-proteins) and subsequently activating the associated downstream signaling cascades. Moreover, they can intercalate into the plasma membrane resulting in an alteration of membrane fluidity and a trigger of a series of cellular responses. (ii) binding with steroid hormone receptors (SHRs) including glucocorticoid receptor (GR), estrogen receptor (ER), progesterone receptor (PR), androgen receptor (AR) and mineralocorticoid receptor (MR) present inside or outside the nucleus by using their hydrophobic backbone is another alternative to trigger downstream cellular responses. Those activated (phosphorylated) SHRs can activate the target molecules through a signaling cascade that brings about various cellular responses. (iii) the ligand-bound SHRs can translocate into the nucleus, where they regulate gene transcription by binding with the specific Response Elements (XRE). This is the so called 'genomic pathway' (indicated by blue arrows). Consequently, the altered gene products can affect the final cellular responses.

Ginsenosides could exhibit their actions through plasma membrane, cytosol, or even in the nucleus. They can initiate their mode of actions by binding to the membrane receptors (e.g. ATPase pump, ion transporters and channels, voltage gated channels and G-proteins) and subsequently activating the associated downstream signaling cascades. As described in the previous section, ginsenosides are amphipathic in nature, they can intercalate into the plasma membrane resulting in the alteration of membrane fluidity and trigger a series of cellular responses. Binding with the intracellular steroid hormone receptors including glucocorticoid receptor (GR), estrogen receptor (ER), progesterone receptor (PR), androgen receptor (AR) and mineralocorticoid receptor (MR), using their hydrophobic 'steroid-like' backbone is another alternative to trigger cellular responses. Like other steroids, ginsenosides can gain access to the nuclear receptors, where they regulate gene transcription by binding with the specific gene response elements to activate the so called 'genomic pathway' [175,176] (Figure 6).

**Figure 7:**
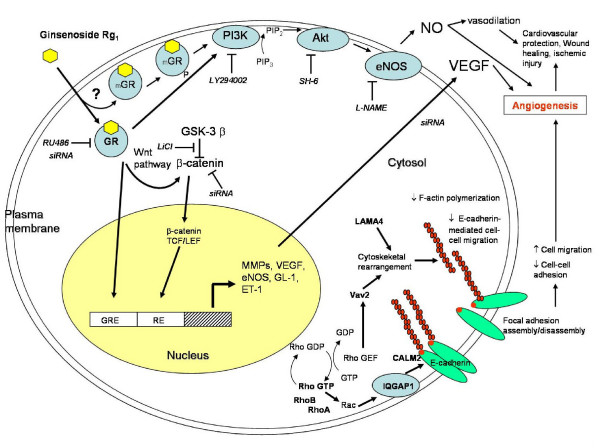
**Schematic overview of ginsenoside Rg_1_-mediated angiogenic action in HUVEC**. Ginsenoside Rg_1_, which acts as a functional ligand of glucocorticoid receptor (GR) (either cytosol GR or membrane-bound GR-mGR), promotes angiogenesis through both non-genomic and genomic pathways. Through the non-genomic pathway, it increases nitric oxide (NO) production via the PI3K-Akt pathway: GR → phosphatidylinositol-3 kinase (PI3K)/Akt pathway → endothelial nitric oxide synthase (eNOS) pathway. Rg_1 _also increases vascular endothelial growth factor (VEGF) production through the GR → PI3K/Akt → GSK3β → β-catenin/TCF pathway. Gene expression profiling data indicated that Rg_1 _could increase the expression of a group of genes (e.g. Rho A, RhoB, IQGAP1, LAMA4, CALM2 and Vav2) which are related to cell-cell adhesion, migration and cytoskeletal remodeling.

A previous study indeed demonstrated that Rg_1 _can trans-activate glucocorticoid response element (GRE)-luciferase activity by acting as a functional ligand of the GR [177]. Glucocorticoids (GCs) have been reported to activate the phosphatidylinositol-3 kinase (PI3K)/Akt pathway after binding with the GR [178]. Interestingly, our recent findings also indicate that Rg_1 _can act as GCs to increase the phosphorylation of GR, PI3K, Akt/PKB and eNOS, leading to an enhancement of NO production in HUVEC (Figure 7). Activation of eNOS was abolished by differential inhibition using RU486 (GR antagonist), LY294002 (PI3K inhibitor) and SH-6 (Akt inhibitor), indicating that Rg_1 _induced NO production from eNOS via the PI3K/Akt pathway. Moreover, knockout of GR by siRNA completely eliminated such reaction. Taken together, we concluded that such NO generation pathway was mediated through the activation of GR by ginsenoside Rg_1 _[179]. This nuclear receptor mediated response, which occurs within seconds or minutes upon stimulation and is independent of any transcriptional activity, is classified as the 'non-genomic' pathway [175,176] (Figure 6).

In a later study, we discovered that the angiogenic Rg_1 _was able to enhance VEGF expression in ECs by increasing the level of β-catenin within the nucleus. We concluded that Rg_1 _induced increase of VEGF in HUVEC through activation of a PI3K/Akt → GSK3β→β-catenin/TCF pathway via GR [180]. In addition, the PI3K/Akt-mediated NO generation has been found in androgen-responsive LNCaP and estrogen-responsive MCF-7 cells by others [181]. Re was found to bind with steroid hormone receptors (including AR, ERα and PR). Lee *et al*., however, showed that ginsenoside Rh_1 _could only activate ER in the CV-1 cell model [173].

The estrogen-like activity of various ginsenosides including Rg_1_, Rh_1 _and Rb_1 _has been reported [170-172]. In fact, ginseng has been used in TCM for alleviation of the symptoms of menopause, which means ginseng, probably ginsenosides, can act as phytoestrogens to elicit an ER-mediated pathway for protecting the cardiovascular system. These recent research findings clearly indicate that ginsenosides may act as steroid hormones to activate various steroid hormone receptors to exert these pleiotropic responses. Although many mechanisms behind the physiological effects induced by ginsenosides are still not clear, their ability to interact with steroid hormone receptors may hold the key to finally elucidate these diverse actions of ginseng.

## Conclusion

At present, ginseng is not only used as a therapeutic by traditional medical practitioners but is also as health supplements readily available in the commercial market. The diversity of highly desirable physiological effects of ginseng has intrigued scientists for years. In general, most of its pharmacological actions have been attributed to a group of triterpenoid saponins called ginsenosides. Although the modulatory effects of ginseng or probably ginsenosides have been extensively investigated, the actual molecular mechanisms remain largely unknown. Recently, it has been found that ginsenosides can act as functional ligands to activate different steroid hormone receptors. Through such mechanisms, ginseng can exert its effects on the human body by acting in a similar way as the steroid hormones. The interaction between ginsenosides and various nuclear steroid hormone receptors may explain the diverse activities of ginseng, which may eventually lead to further development of ginseng-derived therapeutics for angiogenesis-related diseases.

## Abbreviations

2-DE: Two-dimensional gel electrophoresis

AR: Androgen receptor

BCRP: Breast cancer resistance protein

CALM2: Calmodulin

CNS: Central nervous system

ECM: Extracellular matrix

ECs: Endothelial cells

eNOS: Endothelial nitric oxide synthase

ER: Estrogen receptor

FGF: Fibroblast growth factor

GC: Glucocorticoids

GR: Glucocorticoid receptor

GRE: Glucocorticoid responsive element

HDL: High-density-lipoprotein

HUVEC: Human umbilical vein endothelial cells

ICAM-1: Intercellular adhesion molecule-1

IQGAP1: IQ-motif-containing GTPase-activating protein 1

LAMA4: Laminin-α4

LDL: Low-density-lipoprotein

L-NAME: N^ω^-nitro-L-arginine methylester

MDR: Multi-drug resistance

MMPs: Matrix metalloproteinases

MR: Mineralocorticoid receptor

MVD: Microvessel density

MX: Mitoxantrone

OH: Hydroxyl

PA: Plasminogen activators

PAI-1: Plasminogen activator inhibitor-1

Pgp: P-glycoprotein

PKC: Protein kinase C

PPD: Protopanaxadiols

PPT: Protopanaxatriols

PR: Progesterone receptor

RE Responsive element

Rho-GEFs: Guanine nucleotide exchange factors

Rx: Ginsenosides

TCM: Traditional Chinese medicine

TGF-β: Transforming growth factor β

TNF-α: Tumor necrosis factor α

uPA: Urokinase plasminogen activator

uPAR: Urokinase plasminogen activator receptor

VCAM-1: Vascular cell adhesion molecule-1

VEGF: Vascular endothelial growth factor

## Competing interests

The author(s) declare that they have no competing interests.

## Authors' contributions

PYKY conceived and wrote the article and performed the experiments. NKM and TBN drafted the section of 'anti-tumor effects of ginsenosides'. YKC prepared all the chemical structures of ginsenosides. KWL and TPDF conducted the experiments. HWY and RNSW helped to compile the manuscript.
